# Taking a new biomarker into routine use – A perspective from the routine clinical biochemistry laboratory

**DOI:** 10.1002/prca.201000073

**Published:** 2010-12

**Authors:** Catharine Sturgeon, Robert Hill, Glen L Hortin, Douglas Thompson

**Affiliations:** 1 Clinical Biochemistry, Royal Infirmary of EdinburghEdinburgh, UK; 2 Clinical Biochemistry, Sheffield Teaching Hospitals NHS Foundation TrustSheffield, UK; 3 Quest Diagnastics, Inc.Cincinnati, OH, USA; 4 Clinical Biochemistry, St. James' HospitalLeeds, UK

**Keywords:** Analytical performance, Biomarker efficacy, Biomarker evaluation, Clinical biochemistry, Pre-analytical requirements

## Abstract

There is increasing pressure to provide cost-effective healthcare based on “best practice.” Consequently, new biomarkers are only likely to be introduced into routine clinical biochemistry departments if they are supported by a strong evidence base and if the results will improve patient management and outcome. This requires convincing evidence of the benefits of introducing the new test, ideally reflected in fewer hospital admissions, fewer additional investigations and/or fewer clinic visits. Carefully designed audit and cost-benefit studies in relevant patient groups must demonstrate that introducing the biomarker delivers an improved and more effective clinical pathway. From the laboratory perspective, pre-analytical requirements must be thoroughly investigated at an early stage. Good stability of the biomarker in relevant physiological matrices is essential to avoid the need for special processing. Absence of specific timing requirements for sampling and knowledge of the effect of medications that might be used to treat the patients in whom the biomarker will be measured is also highly desirable. Analytically, automation is essential in modern high-throughput clinical laboratories. Assays must therefore be robust, fulfilling standard requirements for linearity on dilution, precision and reproducibility, both within- and between-run. Provision of measurements by a limited number of specialized reference laboratories may be most appropriate, especially when a new biomarker is first introduced into routine practice.

## 1 Introduction

Despite numerous publications on biomarkers – a PubMed search identifies close to half a million articles since 1975 – very few new tests have been added to the routine repertoire of most clinical biochemistry laboratories during that time period. Those that have been introduced on a large-scale include immunoassays for CA125, prostate-specific antigen (PSA) and cardiac troponin. Over the last 15 years, the average rate of introduction of new protein analytes into mainstream clinical laboratories is estimated to have been only 1.5 new proteins *per* year 1. In view of the major expenditure on identifying and characterizing new biomarkers, particularly in the proteomics field, it is desirable to consider why this has been the case – since new technology and diagnostic tests are clearly only of value if used and adopted – and whether there are obstacles that can readily be overcome in order to achieve more timely and efficient introduction of new tests into clinical practice.

Taking a biomarker from the research laboratory successfully into the routine clinical laboratory ideally requires a four-way collaboration, involving the research laboratory (which develops the fundamental concept), the diagnostics industry (which turns the concept into a practical reliable tool), the clinical laboratory (which evaluates the tool in real-life practice) and clinicians (who will help to identify unanswered clinical questions and needs which measurement of a new biomarker might usefully address as well as provide the carefully characterized clinical specimens necessary for its assessment).

Although the decision to introduce a new biomarker will clearly be influenced by different reimbursement policies (or lack of these) and other logistical arrangements in different healthcare systems, the proposed introduction of a new biomarker into routine clinical practice essentially requires rigorous assessment from three different perspectives – those of the clinician, the laboratorian and the healthcare funding organization. In terms of the last, an integrated approach to funding the entire patient-care pathway, including additional tests recommended as parts of other initiatives (*e.g.* Quality Outcome Framework targets in the United Kingdom) rather than piecemeal funding of separate functions (laboratory, pharmacy, radiology, *etc*.) – sometimes termed “silo budgeting”– would clearly be highly desirable but is frequently not in place. In the United States, gaining approval and payment rates for new tests can be a limiting factor in determining whether a new test will be performed 2.

From all three perspectives, it is essential that implementation of a new test should be evidence-based, although other priorities may differ slightly. From the perspective of the healthcare provider, for example, the new test must be cost effective (*e.g.* facilitate admission reduction, decrease length of stay in hospital or replace more expensive testing). From a clinical perspective, a new test must provide information that adds to or replaces information available from existing tests and demonstrably improves patient outcome (*e.g.* selects a cohort of patients likely to benefit from costly drugs). From a laboratory perspective, it must be possible to incorporate the new test readily into the routine workflow (*e.g.* it must have reasonably robust pre-analytical specimen handling requirements), to control the workload post-introduction and of course to fund the test. Funding is perhaps most problematic as laboratories are currently under huge pressure to reduce costs. Regrettably, it is rarely possible to relate expenditure in one area (*e.g.* on calprotectin tests in the laboratory) to resultant savings in another (*e.g.* consequent requirement for fewer much more expensive colonoscopies), although this can be readily demonstrated by economic modeling (British In Vitro Diagnostics Association (BIVDA). BIVDA Manifesto: Unlocking the potential of *in vitro* diagnostics in the National Health Service (NHS). 2010; http://www.bivda.co.uk). In this respect, it is salutary to note that there is evidence of a severe problem of under-utilization of some tests that would potentially benefit diagnosis. This is most likely to occur when laboratories or health systems are operating on a fixed budget and there is major focus on over-utilization of tests, but it should be addressed. Finally, for large-scale implementation of a new test, input and investment from the diagnostics industry is essential. The views of patient representatives may also be helpful in relation both to test acceptability and possible psychological implications.

This article focuses on the practical requirements for successful introduction of a new biomarker from a laboratory perspective (Table 1), highlighting some differences in approach in two fundamentally different healthcare systems, those of the United Kingdom 3 and the United States. (Although the United Kingdom now has four distinct systems for health following devolution in 1999, all four reflect the same basic principles.)

**Table 1:** Key points

(i)	Taking a new biomarker from the research laboratory into the routine clinical laboratory requires proactive three-way collaboration involving the research laboratory, the diagnostics industry and the clinical laboratory.
(ii)	Some tests may be most appropriately offered in specialist laboratories.
(iii)	Rigorous investigation of pre-analytical requirements of a new biomarker is essential at the earliest possible stage of evaluation.
(iv)	Analytical performance must be documented in detail.
(v)	Well-documented evidence of clinical utility and cost-effectiveness in populations representative of those which will be encountered in routine practice is essential for a new biomarker.
(vi)	Evidence is required of the additional diagnostic or predictive information provided by the biomarker when used together with or when replacing other clinical or biochemical tests, *i.e.* its likely beneficial effect on the patient pathway.
(vii)	Appropriate regulatory requirements must be fulfilled.

Some of the requirements for introducing a new biomarker were considered specifically in March 2010 at the “Perspectives in Proteomics” Conference of which this article forms part of this “Focus Issue”, and also at the 12th Bergmeyer Conference 4, which took place the same month under the auspices of the International Federation of Clinical Chemistry and Laboratory Medicine.

## 2 Transition of a new biomarker from research to routine

It is immediately apparent after even superficial review of the relevant literature that many more biomarkers are identified than ever reach routine practice. For the relatively few that do so, the time frame is often years. The tumour marker now known as prostate-specific antigen (PSA) was identified in 1970 5, but it was not until the late 1980s that the first definitive study investigating its clinical utility in prostate cancer was published 6 and another decade later until establishment of the 1st International Standard for PSA 7. As described later in this article, the example of PSA illustrates very well some of the challenges likely to be encountered during the introduction of a new diagnostic test into routine practice. The appropriate clinical application and interpretation of PSA measurements remain controversial even after many years of clinical use of this test.

It is relevant to note that at the time PSA was initially investigated, most routine clinical biochemistry laboratories in the United Kingdom NHS had significant research capability, particularly in developing and optimizing immunoassays. Much of this expertise has since been lost, as recently highlighted in the report of an independent review of NHS Pathology Services, which states that “In the past one of the strengths of the NHS has been the interrelationship between service provision and research” (Lord Carter of Coles. Report of the second phase of the review of NHS pathology services in England 2008; http://www.dh.gov.uk/en/Publicationsandstatistics/Publications/PublicationsPolicyAndGuidance/DH_091985). Relevant to this, a recent Science Council Report observed that “Of special concern to us was the relatively low profile of diagnostic testing and those who undertook this is in the NHS” (The Science Council. Integration and implementation of diagnostic technologies in healthcare. 2004; http://www.sciencecouncil.org).

This reduction in research capacity and lack of visibility, both of which need to be urgently addressed, are among several current barriers to innovation in the clinical laboratory. Increasing focus on service delivery has inevitably meant less time for research activities, even those as relatively straightforward as systematic storage of relevant human biological specimens (http://www.dh.gov.uk/en/Publicationsandstatistics/Publications/PublicationsPolicyAndGuidance/DH_091985). Continual pressure to make cost savings despite a relentlessly increasing workload (frequently 8–10% *per* year, usually without matching budget increases) 8 contributes to the perception that there is no money available for research and the commonly held but erroneous view that research is not an integral part of the remit of a routine clinical laboratory. This view is particularly unfortunate since laboratory staff – who have relevant analytical expertise as well as unparalleled access to clinical specimens – are uniquely well placed to contribute constructively to the evaluation and evidence-based implementation of new tests. Current research needs and expertise in clinical biochemistry may have diminished in the realm of assay development, but there remains critical expertise in the realms of analytical assessment, assay standardization, quality assurance, assessment of reference intervals, physiological variability, optimal specimen collection and processing, correlation with existing tests and interpretation of results, all of which are essential to optimal introduction of a new test. Such needs and expertise apply to test development in the United States as well as in the United Kingdom 2.

Such activity is in accord with increasing appreciation that multidisciplinary input is highly desirable at all stages of clinical evaluation of a new biomarker. Relevant to this, a recent UK government strategy article highlights the importance of research as a core NHS role, particularly when resources are under pressure and there is increasing need to identify new ways of preventing, diagnosing and treating disease 9. How best to direct the funding essential for such activity into laboratory budgets requires careful and innovative thought and should perhaps be considered by the NHS Technology Adoption Centre (NTAC), whose remit includes identifying those technologies which will provide cost effective improved patient outcomes in the NHS (http://www.technologyadoptionhub.nhs.uk/), but which might itself require increased resources to achieve this. Recommendations by the Medical Technologies Advisory Committee (MTAC) of the National Institute of Health and Clinical Excellence (NICE) should inform NTAC decisions as MTAC is responsible for advising on which medical technologies, including diagnostic tests, should be selected for evaluation as well as developing the guidance itself (NICE. New medical technologies programme. 2010; http://www.nice.org.uk/).

The reduction universally observed in test development and research activities represents in part a shift from laboratories making their own reagents and immunoassays to buying most of them from an *in vitro* diagnostics company. This is not an entirely negative development. External quality assessment (EQA)/proficiency testing data clearly demonstrate the benefits of automation, including much improved precision, and there are benefits of scale in centralizing test development processes. Nevertheless, clinical laboratories should play an active role in the final evaluation of assays and in study of their clinical utility.

In considering requirements for successful introduction of new diagnostic tests, it is helpful to review the general criteria that must be met (Table 2), focusing on the roles of both research and specialist laboratories and the somewhat different requirements of high-throughput routine laboratories.

**Table 2:** Requirements for successful transition of a new biomarker from the research environment to routine clinical practice

Requirement	Comment
An unmet clinical need which is clearly understood 33	The purpose of the test should be clear, its use evaluated within a care pathway and its effect on outcome compared directly with existing best practice in the population for which it is intended 35.
Appropriate and well-characterized clinical specimens for both discovery and qualification which mirror the relevant clinical population.	Numerous critical factors must be taken into account when collecting specimens for the studies of new biomarkers, whether for a specific clinical study or for a biobank, as has recently been comprehensively reviewed 14. It is highly desirable that the results of parameters such as albumin, creatinine and CRP should be available for banked specimens to enable interpretation of results and ensure appropriate matching of patient and control samples.
An appropriate and well-validated discovery platform which is robust, reliable and relatively simple to operate.	The importance of using internal standards, identifying measured components, developing standards for calibration and quality control, identifying peaks in spectra and applying established standards for method evaluation have previously been highlighted 36.
Clinical evidence for the biomarker of	Evidence of biomarker-disease association is necessary but not sufficient for effective clinical performance. The critical question is “Do patients undergoing the diagnostic test fare better than similar untested patients?” 37.
• Association with the relevant disease	
• Assessment of clinical utility and impact	
• Circumstances where use of the test would be unjustified	
Rigorous early investigation of pre-analytical factors that might influence interpretation of test results, including the effect of	Quality requirements previously described in detail for tumour marker measurement using immunoassay, mass spectrometry and microarray techniques are relevant to all new tests 23.
• Specimen type, specimen timing and specimen handling	
• Stability in transit and during long-term storage	
• Freeze–thawing	
• Intra-individual biological variation	
• Relevant interventions (*e.g.* biopsy) or medication	
Analytical evidence for the biomarker measurement of acceptable technical performance, including	
• Linearity on dilution	
• Accuracy	
• Precision	
• Reproducibility	
A prototype assay method suitable for early evaluation	Early transfer of a validated biomarker from the research laboratory to a specialist referral laboratory enables confirmation of transferability and assessment in a clinical setting. Some tests may be most appropriately provided by specialist laboratories (as is current practice, *e.g.* for gut hormone screens) with possible later transfer to a high-throughput laboratory.
Transfer of the biomarker to a routine IVD platform, which is only likely if there is	This step represents much greater financial investment than development of the prototype method 33. Introduction of a new test onto an analytical platform may also require modification of existing tests with other implications (*e.g.* for product inserts, *etc*.).
• Convincing evidence of sufficient clinical utility to warrant broad commercial uptake	
• High likelihood that regulatory approval will be granted	
Introduction of the biomarker into the routine clinical laboratory requires	Commissioning diagnostic tests is more sophisticated than simply procurement or contracting procedures. The core of the commissioning process is identification of the clinical need that will be met by the use of the test and the contribution it will make to the patient pathway 20.
• Commissioning the new test because it demonstrably meets an identified clinical need	

### 2.1 Role of the research laboratory

Having selected a promising biomarker for further investigation, it is essential from the earliest stages of evaluation to minimize the risk of introducing methodological bias which may lead to misinterpretation of results 10, 11. This requires considerable care and attention to detail with respect to all phases of analysis when designing studies to be undertaken 12.

#### 2.1.1 Pre-analytical considerations in the research laboratory

Sample collection, processing and storage protocols must be confirmed to be appropriate for each biomarker. Documents describing international efforts to develop best practice have recently been helpfully collated 13. Relevant issues have also been reviewed in more detail, both broadly, in relation to banking of clinical samples for proteomic biomarker studies 14, and more specifically, in relation to criteria for the UK Biobank project 15. The latter is a large prospective study in which samples are being collected from 500 000 participants with the aim of investigating the role of a number of factors in the causes of major diseases of late and middle age. The type of sample, the collection tube and the presence of stabilizers all need to be carefully considered. The storage stability of a potential new biomarker should also be investigated at room temperature, 4 and −30°C, together with the effect of repeated freeze–thaw cycles. Specimen timing may be critical if diurnal variation influences biomarker production *in vivo*, as is the case even for some commonly measured analytes such as cortisol. Early evaluation of intra-individual variation 16 is important. Unless pathological variation of biomarker values is substantially greater than physiological variation from day to day, it is unlikely that the biomarker will be clinically useful. This circumstance also contravenes the major inconvenience and cost of arranging multiple sampling on different days.

Ensuring that specimens are collected strictly according to well-defined protocols at the evaluation stage is essential to minimize the risk of inadvertently introducing subtle differences in sample handling that may affect study results. Selection of clinical specimens for study is, of course, also critical. Appropriate age-matched controls are necessary to minimize the risk of introducing extraneous confounding factors and consequently possible misinterpretation. For example, if evaluating a serum proteomics test for the diagnosis of prostate cancer in a male cohort with prostate cancer and of average age 67 years, it is not appropriate for the comparison group to have an average age 30 years less and include 50% females 10. It is also desirable that the patient cohorts studied resemble as closely as possible the cohort likely to be encountered in routine clinical practice and that the control population is suitably diverse. Confounding pathophysiological factors such as renal dysfunction, hepatic disease, protein-losing disorders, acute-phase responses and nutritional deficits often may influence biomarker levels. For banked specimens, availability of results for basic parameters including serum albumin, creatinine and C-reactive protein (CRP) is therefore highly desirable as these results may be critical to the interpretation of results for a new biomarker. The availability of such information also facilitates appropriate matching of control and patient cohorts during biomarker evaluation. If possible, disease and control specimens should be collected at the same clinics to minimize possible local or procedural differences.

#### 2.1.2 Analytical considerations in the research laboratory

Research laboratories differ from routine clinical laboratories in several important respects. More staff time is likely to be available for relatively complex experimental procedures that are much less feasible in a busy routine laboratory. Early consideration should therefore be given as to whether manual assay procedures could be simplified and ultimately automated if the test is widely adopted. Quality control procedures in a research laboratory may be less rigorous than those in a clinical laboratory but basic steps should be implemented at an early stage to assess reproducibility (both within- and between-batch) and precision at clinically relevant concentrations over a reasonable time period and across different batches of reagents. The lowest reportable concentration, using precision profiles 17, linearity on dilution and recovery of added analyte should also be documented.

#### 2.1.3 Post-analytical considerations in the research laboratory

During early evaluation studies, it is highly desirable that specimens examined are from cohorts of patients similar to those likely to be encountered when using the test in a typical clinical setting. The potential role of the biomarker in clinical practice therefore needs to be considered in the design of early studies of clinical validity, while accepting that this role is likely to be refined as its use increases 18. For example, it was only appreciated some time after free PSA measurement became available that measurement of the free: total PSA ratio is appropriate only when the total serum PSA concentration is within certain limits. Excellent and early collaboration between hospital and research laboratories – whether in academic institutions or in diagnostic companies – should help to ensure that specimens are relevant as well as encourage development of processes that are robust enough for ultimate transfer to routine laboratories. In this respect, the trend in the United Kingdom towards the loss of joint academic and NHS clinical biochemistry departments is unfortunate.

### 2.2 The role of the specialist laboratory

The specialist or referral laboratory can provide a very helpful interim setting during the transition of a new biomarker from research to routine clinical practice. Specialist laboratories such as those forming the United Kingdom Supra-regional Assay Service network (http://www.sas-centre.org/home.html) generally provide assays which meet at least one of the following criteria:

- Small workload (*e.g.* gut hormones)
- Relevant only in rare clinical condition/s (*e.g.* parathyroid hormone-related peptide)
- Technically difficult or requiring specialist equipment (*e.g.* extraction assays for steroids)
- Clinical interpretation complex and requiring specialist expertise (*e.g.* enzyme assays for genetic metabolic disorders such as lysosomal storage diseases)
- In transition from research into routine practice (*e.g.* cytokines)

In principle, evaluation of a new test in a specialist laboratory is attractive for a number of reasons. As well as having the necessary infrastructure (in particular, staff expertise and relatively complex equipment), specialist laboratories receive specimens from numerous other centres and consequently can accrue data on test performance – both analytical and clinical – relatively rapidly. Provided appropriate patient information is collected and collated according to rigorous standards approaching those of clinical trials, evaluation results obtained in the research setting can be confirmed in the specialist laboratory, although this potential could probably be more effectively exploited than has been the case in the past. Specialist laboratories are also very well placed to facilitate and encourage assay standardization, including development of the reference materials and reference methods that are essential to establish both what is being measured and what constitutes an accurate quantitative value 19. These standardization activities would provide a sound basis – underpinning well-documented quality objectives and helping to reduce delays such as those encountered previously for PSA 7 and other analytes – for the transfer of a new test to multiple platforms prior to implementation in the routine clinical laboratory.

### 2.3 Taking a new biomarker into the routine clinical laboratory

Critical requirements for introducing a new biomarker into the routine clinical laboratory are perhaps best considered under three broad headings, *i.e.* those requirements reflecting broad overall policy (including funding arrangements and the need for user education), those related to logistical requirements within the laboratory, and those related to the involvement of diagnostics companies in making the new biomarker available on an automated analyzer. For all three, early discussion between relevant clinical, laboratory and/or diagnostic company staff is highly desirable. In the past, this has most often relied on personal or institutional contacts but increasingly more formal arrangements are being developed. These also provide an excellent opportunity to encourage early attention to ensuring quality assurance, development of reference materials and assay standardization. A prototype example is the Biomarkers Consortium, a major public–private biomedical research partnership of 24 companies and 30 non-profit organizations which is managed by the Foundation for National Institutes of Health with the aim of encouraging the effective identification and deployment of biomarkers (Foundation for the National Institutes of Health: The Biomarkers Consortium. 2010; http://www.fnih.org/work/key-initiatives/biomarkers-consortium).

#### 2.3.1 Pre-requisites for success in bringing a new biomarker into routine practice – a broad policy perspective

Increasing pressure to demonstrate that healthcare is both evidence based and cost effective means that, without convincing evidence that a proposed new test will have a beneficial effect on patient outcome, a new biomarker is unlikely to be introduced in any publicly funded healthcare system 20, 21. It is essential that evidence supporting introduction of a new test is clearly presented, comprehensive and independently confirmed in at least two centres. Such an approach should address the reluctance some laboratories may have to accept evaluation data from other sources, a view that can result in repetition of the same study. This should be unnecessary if the original studies are well designed, appropriately designed and well documented although verification of performance (which is fundamentally different) 22 is appropriate when introducing a new test routinely. Formal recommendations from NICE or NTAC could provide additional assurance of the validity of evaluation data.

A business case or plan for funding a proposed new test is essential, and very careful consideration must also be given to the workload implications of making the test available and how best to manage demand.

#### 2.3.2 Pre-requisites for success in bringing a new biomarker into routine practice – regulatory aspects

A significant issue in the development cost of a new biomarker – and one which also may contribute to delay in its reaching clinical use – is the need to meet requirements for regulatory approval in Europe and/or the United States. These differ, with current European requirements perhaps tending to focus more on analytical validation and those in the United States on clinical validity. The European In Vitro Diagnostic Medical Devices Directive, which in its Appendix 1 defines minimum requirements that must be met by *in vitro* diagnostic devices, is currently being revised and significant tightening of the requirements is anticipated (http://www.bivda.co.uk). It is neverthless helpful to consider current requirements which undoubtedly influence how rapidly a new test is introduced.

In Europe, the application of a CE mark by the manufacturer (or their authorized agent) means that the diagnostic meets the requirements of the Directive and is therefore permitted to be placed on all European markets. This relies on self-certification by the manufacturer supported by relevant technical data. Requirements are more stringent for some tests (*e.g.* blood grouping, HIV and PSA) and a Notified Body must be involved. More recent test technologies, developed since the last revision of the Directive, while within its scope do not fit well within the current Directive requirements.

In the United States, there are three pathways to test approval. The simplest pathway is for laboratory-developed (or home brew) tests, which must meet specified regulatory requirements for analytical validation, quality control, external validation, personnel qualifications, training and documentation under the Clinical Laboratory Improvement Amendment federal regulations. Usually, these tests are not reviewed by the Food and Drug Administration. The second (510 k) pathway is for clearance of commercially available assays and involves a comparison and some evidence of clinical comparability with an existing cleared or approved test. The third pathway is pre-market approval, which requires a clinical trial and evidence of adequate clinical performance. This is required for devices considered to be in high-risk categories (*e.g.* tumour markers and HIV tests). There is a high cost for assuring regulatory compliance in the United States, which is reflected in the different test menus for automated analyzers marketed in both the United States and the European Union. There are a considerable number of tests that are not available in the United States and often a several year lag between availability of a test in the United States and Europe. The approach in the United States is perhaps more difficult to apply rigorously, because measures of clinical sensitivity and specificity depend substantially on the population tested and are not entirely a characteristic of the test. It is often forgotten that claimed figures for clinical sensitivity and specificity should be qualified with the statement that these were derived in a specific population of well-characterized subjects and may not apply for patients with different characteristics.

#### 2.3.3 Assessing the impact of a new biomarker on patient outcome

A new biomarker is only likely to be useful if three circumstances apply: (i) that the biomarker results are appropriate precisely for the required application, (ii) that the marker results separate patients into two or more populations whose outcomes differ so significantly that clinicians would treat the two groups differently and (iii) that the divergence of outcomes for patients stratified for treatment according to their biomarker results is reliable 23. Availability of effective treatment interventions is usually an essential pre-requisite for improving outcome.

Demonstrating the effect of biomarker measurement on patient outcome is complex for a number of reasons. As has recently been elegantly described, clinicians use biomarker results (*e.g.* troponin) together with physical observations (*e.g.* symptoms of a coronary event) to decide whether to initiate further intervention (*e.g.* cardiac catheterization) which may improve future outcome (*e.g.* mortality rate) 24. It is clearly difficult to differentiate the many contributory variables – which are likely to be influenced not only by study design but also by the patient population studied 25 – and a major problem in demonstrating effect is the remoteness of outcome from the test result 24. In this context, multivariate analysis is essential. By taking into account the contributions of other biomarkers or clinical factors, multivariate analysis allows an assessment of the extra predictive effect of adding a novel marker to the current clinical and biochemical diagnostic tools used in a particular disease or pre-disease state. It is instructive to discover that, despite many articles describing the diagnostic performance of serum Heart-type Fatty Acid Binding Protein since its proposal as a marker of Acute Coronary Syndrome (ACS) in 1988, multivariate analysis in patients suspected of ACS has been published only as recently as 2010 26.

Intelligently designed randomized controlled trials, which adhere to the recommendations of the CONSORT statement 24, 27 and the REMARK guidelines 25 are likely to become more widely used in the early assessment of new biomarkers. Modeling studies may cost-effectively complement such trials as they permit simulation of the effect of testing in much larger populations than can be achieved in clinical trials. Appropriate information both about the diagnostic accuracy of the test and about the effect of consequent treatment decisions in a well-defined patient group is essential for modeling studies. Such studies can be particularly helpful in defining required parameters of analytical performance by assessment of the effect of bias and imprecision, as has been demonstrated for measurement of PSA in a screening context 28 and for use of glucose meters in patients on intensive insulin treatment 29.

Modeling studies can readily provide valuable insight into analytical criteria that must be met, inform development of appropriate protocols and predict the downstream consequences of testing. However, they are less likely to be able to provide information about clinical outcomes including morbidity, mortality, length of stay in hospital or readmission rates, all of which are best assessed through carefully designed audit studies.

Proposed introduction of a new biomarker will in some cases be supported by guideline recommendations, but in practice this is more likely to be the case when the biomarker is already reasonably well established.

#### 2.3.4 Development of a business case for funding a new biomarker

The cost of biomarker analysis is generally much less than that associated with therapeutic interventions or other diagnostic procedures such as magnetic resonance imaging and is likely to represent only a fraction of the cost of a patient episode. Cost studies have therefore often not been considered when the biomarker is one small cog in an overall treatment plan 21. Nevertheless, from a laboratory viewpoint, ensuring that an appropriate reimbursement mechanism is in place is essential before introducing a new biomarker. This minimizes the risk of the laboratory ultimately carrying the costs for unfunded tests upon which clinicians have come to rely, as has occasionally happened in the past. How the business case is prepared will depend on local, regional and/or national funding arrangements and should include consideration of how introduction of the new test would lead to cost savings elsewhere in the health system (*e.g.* as reflected in fewer admissions to hospital, decreased requirement for more expensive radiological studies, more efficacious prescribing of medication). One of the best examples is in breast cancer, where relatively inexpensive measurement of HER2 status ensures that the costly companion drug Herceptin® is only prescribed to those patients likely to benefit. The annual cost for a UK patient on Herceptin® has been estimated by BIVDA to be £20 000 *per* year, whereas the cost for the screening test to identify HER2 positive patients is about £225 and the initial screen using immunochemistry is much less than this (http://www.bivda.co.uk).

If business cases for new tests were developed regionally or nationally under the auspices of NTAC, the Department of Health, or another funding body (after careful consideration of relevant evidence including the predicted impact of the new test on the patient pathway), and reviewed centrally, this could be more efficient than current practice, as well as encouraging more uniform introduction of the test.

#### 2.3.5 Managing demand for a newly introduced test

Although 70–80% of all healthcare decisions affecting diagnosis or treatment involve a pathology investigation 30, it is also the case that over-requesting of many biochemistry tests is widespread, with estimates varying from 25–40% to up to 98% in one study (http://www.dh.gov.uk/en/Publicationsandstatistics/Publications/PublicationsPolicyAndGuidance/DH_091985). It has recently been suggested that eliminating inappropriate testing could save the NHS up to £1 billion *per* annum in test costs alone 8. Although major efforts are now being made to address this retrospectively for established tests 8, introducing a new biomarker – whether to replace a previous test (*e.g.* troponin replaced lactate dehydrogenase isoenzyme and also to some extent creatine kinase-MB isoenzyme measurements), or to introduce a new parameter (*e.g.* brain natriuretic peptide) – provides a unique opportunity to implement appropriate requesting patterns from the beginning. This was achieved with some success when troponin testing was introduced, when many hospitals limited the test to carefully defined patient groups and often required consultant approval for requests. Other approaches have also been described 8, and such activity, which is integral to service development and improvement, should be given high priority when introducing a new biomarker. An effective means of educating users about requesting, preferably electronically at the time the request is made, is almost certainly the most vital component for success. It is of course also essential to ensure that all tests that the new biomarker should replace are fully withdrawn. Some approaches that may encourage adoption of new practice (*e.g.* not ordering radiological scans when replaced by a new biomarker) have been previously described 31.

### 2.4 Pre-requisites for success in bringing a new biomarker into routine practice – a broad logistical perspective

In order to consider the pre-analytical and analytical requirements for efficiently introducing a new biomarker into the routine high-throughput laboratory, it is essential to have an appreciation of how such laboratories operate. Table 3 summarizes some relevant figures for a typical UK teaching hospital which is one of three adult hospitals serving a population of approximately 1 million people. The figures show the typically high throughput of a modern clinical biochemistry laboratory, where any requirement for special handling outwith the established workflow is likely to be problematic.

**Table 3:** Workload figures for a typical UK acute teaching hospital clinical biochemistry laboratory

Statistic	Number
Population served	750 000
Number of beds	800
Number of samples/year	750 000a)
Number of samples/day	2200
Number of samples/hour at peak time (between 2 and 6 pm on weekdays)	500

a) Representing 5.0 million tests requested in total.

#### 2.4.1 Pre-analytical considerations in the routine laboratory

Numerous different types of specimen – primarily blood and urine but also cerebrospinal or pancreatic fluids, semen, microbiological swabs and others – arrive at the laboratory reception desk, where are they sorted according to the test requirements for processing and storage. Specimens are usually bar coded during the booking-in process, at which time patient details and the tests required are entered into the laboratory computer. Increasingly, many of these processes are at least partially automated. Although specimens from within the hospital may be delivered by porters or through pneumatic tube systems from ward or clinic to the laboratory, those from other hospitals or general practice arrive by van and hence the delay from time of sampling to processing may exceed 16 h.

This means that ideally a new biomarker should be measurable in blood, serum or urine, should be reasonably robust and should not require special handling or storage in transit to the laboratory. The likelihood of having to reject specimens as unsuitable tends to be higher if there are special requirements, *e.g.* having to separate and freeze plasma specimens for adrenocorticotropic hormone within 15 min of collection.

As discussed previously, it is very helpful if there are no specific timing requirements or a need for multiple samples on different days. Even for well-established tests such as testosterone in males or progesterone in females, failure of requestors to ensure that specimens are timed appropriately (*i.e.* between 8 and 11 am or 7 days prior to the next expected menstrual cycle respectively) means that results obtained are often meaningless and a significant number of tests have to be repeated, at additional cost as well as inconvenience to the patient. Early awareness of potential effects on results for a new biomarker of relevant medications, illness or intervention (*e.g.* respectively, oral contraceptive medication on luteinizing hormone, non-thyroidal illness on thyroid hormones or prostatic biopsy on PSA) is also highly desirable although inevitably some caveats are only likely to be identified with increasing use of the test.

Indiscriminate test requesting without first considering carefully whether the result is likely to be helpful or relevant is always undesirable. Clear and specific information about the clinical circumstances in which a new biomarker is to be used should therefore be widely and proactively disseminated – in advance of the test being made available as well as during and after its introduction. Early inclusion of details of a new test in existing information resources such as Lab Tests On Line (http://www.labtestsonline.org.uk/) and the National Library of Medicine Catalogue (http://nlmc.x-labsystems.co.uk/) is highly desirable.

**Figure 1:**
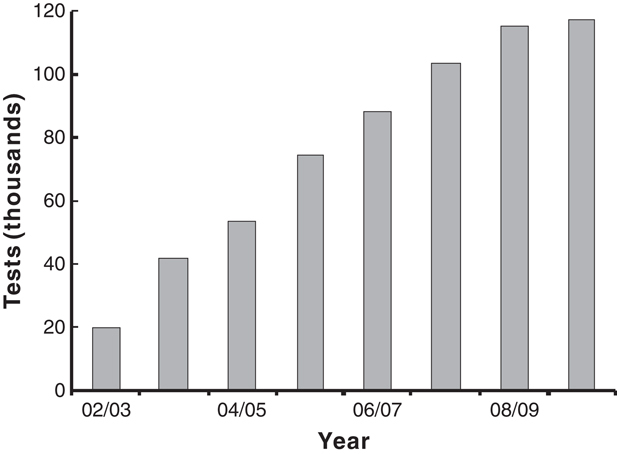
Effect on workload of transferring measurement of CRP from a specialist to a routine laboratory.

Unexpected over-requesting will of course affect cost analyses undertaken prior to introduction of the test, a problem that can also occur when a test previously offered only by specialist referral laboratories is made available in a routine laboratory. Figure 1 shows what happened in one hospital when measurement of C-reactive protein (CRP), an acute phase reactant, was transferred from a unit in which the turn-around time (sample receipt to result issue) was 2 wk to a clinical biochemistry department which provided a turn-around time of 20 min. Although the move had been predicted to be cost neutral, as a consequence of the much improved service provided (including availability of the test on the clinical biochemistry request form), the workload increased nearly sevenfold over as many years and the cost of providing CRP measurements is now the highest of any single test in the laboratory. Since it is unlikely that the clinical need for CRP measurement has increased to this extent in a laboratory where use of ultrasensitive CRP is not being promoted, the main cause is almost certainly inappropriate requesting, a problem that has been encountered elsewhere for CRP, although somewhat less dramatically 32. In the latter study, strategies to control demand were introduced and successfully reduced the number of requests from acute admission units.

#### 2.4.2 Analytical considerations in the routine laboratory

As a consequence of the high workload and perceived need for rapid turn-around time in routine clinical biochemistry laboratories, assay automation is essential for almost every test. There is little staff time for manual assays or complex trouble-shooting and – as during the pre-analytical phase – any complicated processes are likely to be difficult to incorporate. This may be exacerbated in the future by proposed workforce changes in the United Kingdom which may lead to operation of automated analyzers by less highly trained staff.

As summarized in Table 2, a new biomarker must, of course, fulfill standard quality requirements for good performance, including those of relevant regulatory authorities. Prior to providing results for clinical specimens, verification studies to confirm that the method is performing as expected should be undertaken following a defined protocol such as that developed by the UK Association for Clinical Biochemistry 22.

Robust internal quality control procedures must also be in place. EQA is not likely to be available until a reasonable number of laboratories (often a minimum of ten) offer the test. In the absence of an EQA or proficiency testing scheme, informal exchange of samples among laboratories offering the test can provide some assurance that results are similar and may highlight potential difficulties at an early stage, when it is relatively easy to address them. Exchanging information about possible clinically relevant interferences and other aspects of best practice – including appropriate reference intervals, decision limits and interpretation – is also very helpful.

#### 2.4.3 Post-analytical considerations in the routine laboratory

Appropriate reference interval data should be readily available from the laboratory together with clear guidance about clinical interpretation of results in relevant patient groups. This is particularly important for a newly introduced biomarker since clinical staff will not be familiar with the new test and its limitations. Laboratory staff can play a major role in collecting audit data required to assess test performance in routine clinical practice. Recording any unexpected or atypical results and discussing these at an early stage with clinical colleagues is also highly desirable. Effective clinical audit studies should also be conducted to evaluate whether introduction of the new test has met expectations and to identify any problems at an early stage.

### 2.5 Pre-requisites for success in bringing a new biomarker into routine practice – automation and the diagnostics industry

As indicated above, automation is essential in modern high-throughput laboratories. Operational procedures are relatively straightforward and involve maintaining the analyzer and checking internal quality control specimens several times daily, according to the manufacturers' instructions and strictly defined protocols. Reagents are kept on board the machine and specimens are loaded as they arrive. The analyzer reads the bar code on each specimen and determines which tests are to be done. Once results have been technically validated by the operator, they are automatically downloaded to the laboratory computer for clinical authorization and before being uploaded to the hospital information system.

Major analytical advantages of automated analyzers as compared with manual assays include the convenience and speed of analysis (*e.g.* test results can be available at any time of day or night, often within 20 min), the possibility of assaying samples as they arrive (*i.e.* “random access”) rather than in batches, and the excellent precision achievable (*i.e.* superior to that achievable with manual pipetting). Logistically, their relative simplicity means that all staff can readily be trained to use the analyzer, bar-coded primary tubes can be used to minimize the risk of error, and capacity is not an issue.

When considering introducing a new biomarker to the routine laboratory, there are additional issues associated with assays that rely on other technologies such as mass spectrometry as well as with immunoassay methods which require transfer to an automated platform.

#### 2.5.1 Mass spectrometry in the routine clinical biochemistry laboratory

There is ever-increasing interest in clinical biochemistry laboratories in introducing mass spectrometric procedures for established analytes including steroids, vitamin D and therapeutic drugs such as cyclosporine and tacrolimus. These analytes are difficult to measure reliably by immunoassay methods, which tend to be less specific and more prone to interference than mass spectrometric techniques. The major drivers for replacing immunoassay with the latter are increased analytical quality and decreased cost, as the savings on immunoassay reagents can rapidly recoup the initial purchase price of a mass spectrometer.

Although availability of mass spectrometry in routine laboratories is attractive when considering introducing a new biomarker developed on such a system, it is important to be aware of some caveats. It is unlikely to be feasible to train all staff in this specialized technique. In one clinical laboratory offering a tacrolimus service by mass spectrometry, only 30% of staff are trained to run the instrument as compared with all staff for the automated immunoassay analyzer previously used for tacrolimus. In the same laboratory, capacity is an issue and the instrument requires a dedicated member of staff for the whole day to complete the workload, *i.e.* much more hands-on time than required for the immunoassay analyzer, which is essential a “walk-away” instrument. These issues make staff deployment more complicated. In addition, relatively complex sample manipulation is required, some of which is not in bar-coded tubes, making sample handling errors more likely. Accurate pipetting, a skill which unfortunately can no longer be assumed in some highly automated laboratories, is also essential. Until mass spectrometric procedures can be simplified considerably and ideally automated, immunoassay is likely to continue to have a major role in routine laboratories.

#### 2.5.2 Transfer of new biomarkers onto automated analyzers

From a commercial perspective, transfer of a new biomarker onto an existing analytical platform requires significant financial commitment and is wholly dependent on the conviction of at least one diagnostics company that the test will become widely used. This requires not only that all the test characteristics previously described are satisfactorily met but also that the development costs of automation will ultimately be recouped and provision of the test will be financially profitable. Some reworking of the test may be required to ensure that it runs optimally on the platform 33 and provides results identical to those obtained with earlier manual assays. As discussed above, regulatory aspects are critical and a clinical trial using the assay in its final form is usually necessary 33.

## 3 Future prospects

New biomarkers can be taken from research into routine practice provided there is sound evidence of clinical utility, funding can be assured, mechanisms are in place to ensure that the test is done only for those likely to benefit, analytical procedures are simple and robust, and quality is verified through internal quality control and EQA/proficiency testing procedures. For these requirements to be met in a timely manner for a specific biomarker, it is necessary to learn from past mistakes and perhaps to think differently in the future.

Exemplifying the need to learn from past mistakes, an early audit following introduction of troponin testing in a UK hospital, for example, demonstrated no advantage to patients or the NHS of the test in selecting patients for further cardiac investigation and/or reducing length of stay 34. Reasons identified for this most unexpected conclusion were simple. As reagents were not stable enough to allow continuous access of samples, they were run in batches and the intended turn-around time was not met in 29% of cases. In addition, 80 of 109 requests had no information regarding timing of the blood sample in relation to the clinical indication and no indication was even given for 39% of requests. As described above, this illustrates how considerable care and attention to detail is required at every stage of evaluation, development and ultimately routine measurement of a new biomarker.

For the future, much improved involvement and collaboration of all interested parties – including experts in discovery and assay development, in health policy, in clinical trial units, in the diagnostics industry and in laboratories responsible for providing clinical testing – will almost certainly lead to earlier identification and implementation of promising new biomarkers.

In a unique prototype project funded by the UK NHS National Institute of Health Research, the possibilities of such collaboration are currently being explored with the aim of developing a rigorous evidence-based approach to protein biomarker evaluation (Biomarker evaluation and translation. An NHS National Institute of Health Research programme in renal and liver diseases. 2010; http://www.biomarkerpipeline.org/nihr/programme.html). The programme is made up of three closely related and interdependent strands. In the first strand, a multi-disciplinary team of health methodologists and statisticians are undertaking modelling studies to define aspects of best practice in evaluation new tests. In the second analytical strand, clinical scientists are reviewing and identifying potential biomarkers, developing ELISAs for rapid preliminary evaluation of their clinical potential, and then undertaking more rigorous assessment of the most promising markers using well-characterized banked specimens. In the third strand, a randomized controlled trial is being conducted to assess the clinical value to the NHS of a panel of biomarkers that will be available on a commercial platform. Although there are inevitably some challenging issues, this approach may provide a helpful prototype for more rapid introduction of useful new tests into the routine clinical laboratory.

## Abbreviations

BIVDA: British In Vitro Diagnostics Association
CRP: C-reactive protein
EQA: external quality assessment
NHS: National Health Service
NICE: National Institute of Health and Clinical Excellence
NTAC: NHS Technology Adoption Centre
PSA: prostate-specific antigen
